# Airway Microbial Community Turnover Differs by BPD Severity in Ventilated Preterm Infants

**DOI:** 10.1371/journal.pone.0170120

**Published:** 2017-01-27

**Authors:** Brandie D. Wagner, Marci K. Sontag, J. Kirk Harris, Joshua I. Miller, Lindsey Morrow, Charles E. Robertson, Mark Stephens, Brenda B. Poindexter, Steven H. Abman, Peter M. Mourani

**Affiliations:** 1 Department of Biostatistics, Colorado School of Public Health, University of Colorado, Aurora, Colorado, United States of America; 2 Section of Pulmonary, Department of Pediatrics, School of Medicine, University of Colorado, Aurora, Colorado, United States of America; 3 Department of Epidemiology, Colorado School of Public Health, University of Colorado Denver, Aurora, Colorado, United States of America; 4 Section of Infectious Disease, Department of Medicine, School of Medicine, University of Colorado, Aurora, Colorado, United States of America; 5 Perinatal Institute, Cincinnati Children’s Hospital Medical Center, Cincinnati, Ohio, United States of America; 6 The Pediatric Heart-Lung Center, Department of Pediatrics, School of Medicine, University of Colorado, Aurora, Colorado, United States of America; 7 Section of Critical Care Medicine, Department of Pediatrics, School of Medicine, University of Colorado, Aurora, Colorado, United States of America; Forschungszentrum Borstel Leibniz-Zentrum fur Medizin und Biowissenschaften, GERMANY

## Abstract

Preterm birth exposes the developing lung to an environment with direct exposure to bacteria, often facilitated by endotracheal intubation. Despite evidence linking bacterial infections to the pathogenesis of bronchopulmonary dysplasia (BPD), systematic studies of airway microbiota are limited. The objective was to identify specific patterns of the early respiratory tract microbiome from tracheal aspirates of mechanically ventilated preterm infants that are associated with the development and severity of BPD. Infants with gestational age ≤34 weeks, and birth weight 500–1250g were prospectively enrolled. Mechanically ventilated infants had tracheal aspirate samples collected at enrollment, 7, 14, and 21 days of age. BPD was determined by modified NIH criteria with oxygen reduction tests; infants without BPD were excluded due to low numbers. Aspirates were processed for bacterial identification by 16S rRNA sequencing, and bacterial load by qPCR. Cross-sectional analysis was performed using 7 day samples and longitudinal analysis was performed from subjects with at least 2 aspirates. Microbiome analysis was performed on tracheal aspirates from 152 infants (51, 49, and 52 with mild, moderate, and severe BPD, respectively). Seventy-nine of the infants were included in the cross-sectional analysis and 94 in the longitudinal. Shannon Diversity, bacterial load, and relative abundance of individual taxa were not strongly associated with BPD status. Longitudinal analysis revealed that preterm infants who eventually developed severe BPD exhibited greater bacterial community turnover with age, acquired less *Staphylococcus* in the first days after birth, and had higher initial relative abundance of *Ureaplasma*. In conclusion, longitudinal changes in the airway microbial communities of mechanically ventilated preterm infants may be associated with BPD severity, whereas cross-sectional analysis of airway ecology at 7 days of age did not reveal an association with BPD severity. Further evaluation is necessary to determine whether the observed longitudinal changes are causal or in response to clinical management or other factors that lead to BPD.

## Introduction

Infants born preterm are at high risk for developing bronchopulmonary dysplasia (BPD), a chronic lung disease of prematurity. BPD is characterized by prolonged need for oxygen therapy, frequent pulmonary infections requiring hospitalizations, asthma, exercise intolerance, and pulmonary hypertension [1, 2]. BPD results from the adverse effects of early exposure of the lung to the extrauterine environment, as well as, the need for supportive interventions such as mechanical ventilation and high levels of oxygen delivery [3–5]. These stimuli can lead to inflammation of the immature lung that disrupt normal lung development [6]. Thus, further understanding of risk factors and mechanisms that promote inflammation and impair lung development after preterm birth is critical to instituting better prevention and treatment strategies.

Past laboratory and clinical studies have clearly shown that inflammatory responses to adverse environmental stimuli can interfere with lung growth after preterm birth [7, 8]. While antenatal and postnatal infections, hyperoxia, and mechanical ventilation have garnered much attention as inflammation provoking stimuli [9], the possible impact of airway colonization and invasion on the development of BPD is less clear with few studies examining this question [10, 11]. It has been shown that bacterial exposures even without overt infection may influence maturation of the immune system, which in turn, modulates lung development and the response to later infections. The presence of *Ureaplasma* in the respiratory tract of preterm infants, even without signs of infection, has been associated with a pro-inflammatory response and an increased risk for BPD [12]. Thus, postnatal exposure to colonizing bacteria, may impact the preterm infant’s immune response and subsequent lung development.

Recent evidence has revealed that normal lungs contain diverse microbial populations, called microbiota [13–16]. However, standard bacterial culture methods cannot effectively identify the full spectrum of organisms living within the respiratory tract and are inefficient for these investigations. Molecular detection of the genetic blueprint of the microbiota can, in an unbiased manner, identify bacteria in a specimen enabling comprehensive evaluation of microbiome-phenotype associations [10, 17–19]. Study of the respiratory tract microbiome relative to disease outcomes is a novel approach to further link microbial-host interactions to the pathogenesis of chronic lung disease of prematurity.

Ecological shifts in the microbiome, characterized by reduction in microbial diversity and domination by potentially pathogenic bacteria, are associated with augmented inflammatory responses and increased risk for infection and other immune mediated diseases. Similar ecological shifts in the lungs of patients with cystic fibrosis (CF), asthma, and chronic obstructive pulmonary disease (COPD) are associated with worsening lung disease [20–24]. Therefore, we hypothesized that specific patterns of the early respiratory tract microbiome in mechanically ventilated preterm infants (bacterial load and diversity) would be associated with the development of BPD and its severity. We tested this hypothesis two ways: 1) in a cross-sectional analysis of tracheal aspirate specimens collected at 7 days of age from mechanically ventilated preterm infants and 2) in a longitudinal analysis evaluating the change in the airway microbiome in mechanically ventilated preterm infants who had at least 2 tracheal aspirates collected in the first 3 weeks after birth.

## Methods

### Study design and subjects

**Fig 1:**
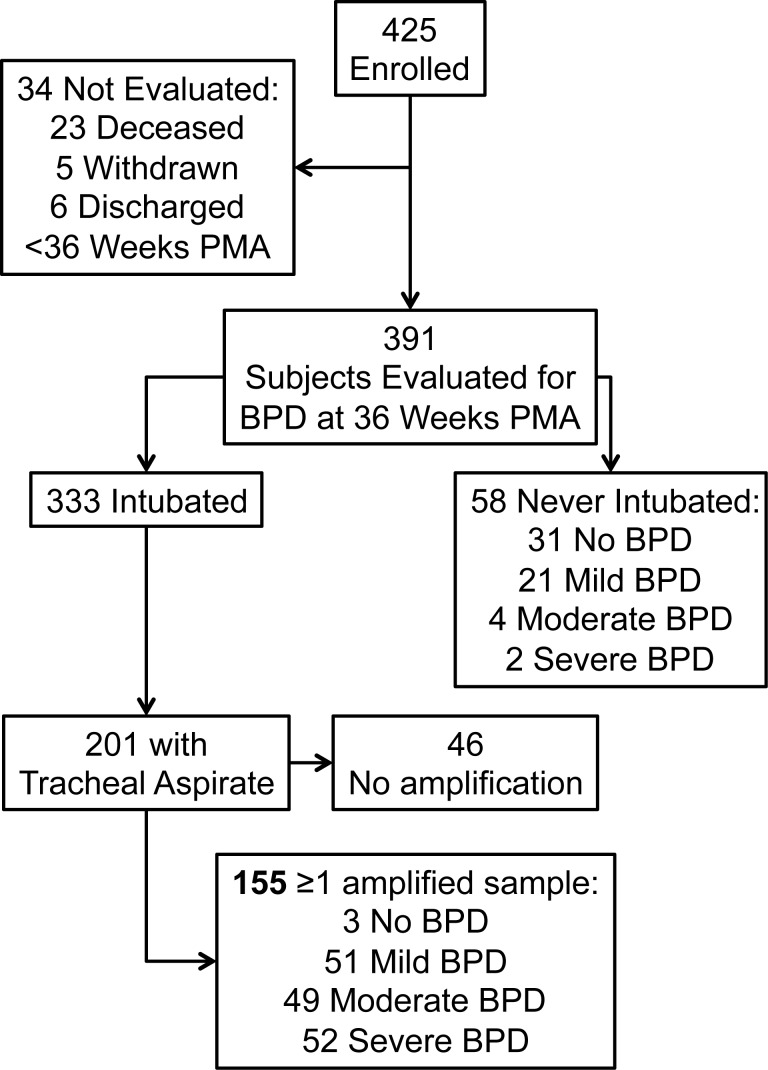
Enrollment, tracheal aspirate collection, and BPD status of study participants.

The patient specimens and data for this study were obtained from a two-center (the University of Colorado School of Medicine, Anschutz Campus and Indiana University School of Medicine) observational longitudinal study that enrolled a cohort of preterm infants between July 2006 and February 2013. The protocol was approved by the Institutional Review Boards at each of the participating sites, and written informed consent was received from the parents or guardians of all participants. Infants with gestational age at birth less than 34 weeks and birth weight between 500 and 1250 g were screened for enrollment. Exclusion criteria included clinical evidence of congenital heart disease (except patent ductus arteriosus [PDA], patent foramen ovale [PFO] or atrial septal defect [ASD] < 1cm, or ventricular septal defect [VSD] < 2mm if known prior to enrollment); lethal congenital abnormality; and futile cases (anticipated death prior to hospital discharge). Subjects were required to be enrolled within 7 days of age. Infants who required mechanical ventilation had tracheal aspirates samples collected at enrollment, 7, 14, and 21 days of age (+/- 48 hours). Infants who were mechanically ventilated and had at least one tracheal aspirate collected were included in this study. Tracheal aspirates collected closest to 7 days of age but within 5–9 days were used for cross-sectional analyses, and infants with at least two tracheal aspirates collected were included in the longitudinal analysis. Infants who died, withdrew, or were transferred or discharged without undergoing BPD assessment at 36 weeks PMA were also excluded (Fig 1). Detailed clinical data including maternal and birth history, hospital course and physiological assessments were collected. BPD status and severity was assessed at 36 weeks PMA using a modification of the NIH workshop definition [5] with application of the oxygen reduction test as described by Walsh [25] and described previously [26].

### Specimen collection and processing

Tracheal aspirate specimens were collected as described in detail previously [10]. Briefly, 0.5 mL of sterile 0.9% saline (without preservative) was instilled via the endotracheal tube (ETT) followed by 3–5 ventilator breaths. A 5 or 6 French sterile suction catheter was inserted to 0.5cm below the end of the ETT and secretions were suctioned into an attached sterile specimen trap as the catheter was slowly withdrawn. Specimens were then centrifuged at 250g for 20 minutes at 4°C. The supernatant was removed and the pellet was stored at -70°C until microbiome analysis.

### Laboratory assays

#### Quantitative PCR

DNA extractions were performed using the Qiagen EZ1 Advanced automated extraction platform (Qiagen Inc., Valencia, CA) with the bacterial card and tissue extraction kit. All sample manipulation was done in the BSL2 hood with appropriate laminar flow. Frozen samples were thawed at 4°C and vortexed to ensure mixing. An aliquot of 200 μl for extraction was transferred into the tube provided with the EZ1 kit. Remaining sample was stored at -70 C. Extraction reagent cartridges, elution tubes and tip holders were loaded into the EZ1 sample rack as instructed by the manufacturer. Elution volume of 100μl was selected and EZ1 DNA Tissue Kit program was run. Elution tubes with DNA extract were stored at -20°C. DNA extraction reagents were confirmed free of bacterial DNA by performing control extractions utilizing buffer or PCR grade water. Total bacterial load was measured using a quantitative real-time PCR assay that has been previously published [27, 28].

#### Sequencing

Bacterial profiles were determined by broad-range amplification and sequence analysis of 16S rRNA genes following our previously described methods [29, 30]. In brief, amplicons were generated using primers that target approximately 300 base pairs of the V1/V2 variable region of the 16S rRNA gene. *PCR* products were normalized using agarose gel densitometry, pooled in approximately equimolar amounts, gel purified, and concentrated using a DNA Clean and Concentrator Kit (Zymo, Irvine, CA). Pooled amplicons were quantified using Qubit Fluorometer 2.0 (Invitrogen, Carlsbad, CA). The pool was diluted to 4nM and denatured with 0.2 N NaOH at room temperature. The denatured DNA was diluted to 20pM and spiked with 10% of the Illumina PhiX control DNA prior to loading the sequencer. Illumina paired-end sequencing was performed on the MiSeq platform using a 500 cycle version 2 reagent kit.

### Analysis of Illumina paired-end reads

As previously described, Illumina MiSeq paired-end sequences were sorted by sample via barcodes in the paired reads with a python script[29]. Sorted paired end sequence data were deposited in the NCBI Short Read Archive under accession number SRP066782. The sorted paired reads were assembled using phrap [31, 32]. Pairs that did not assemble were discarded. Assembled sequence ends were trimmed over a moving window of 5 nucleotides until average quality met or exceeded 20. Trimmed sequences with more than 1 ambiguity or shorter than 200 nt were discarded. Potential chimeras identified with Uchime (usearch6.0.203_i86linux32) [33] using the Schloss [34] Silva reference sequences were removed from subsequent analyses. Assembled sequences were aligned and classified with SINA (1.2.11) [35] using the 629,125 bacterial sequences in Silva 111[36] as reference configured to yield the Silva taxonomy. Operational taxonomic units (OTUs) were produced by clustering sequences with identical taxonomic assignments. This process generated 22,512,493 sequences for 350 samples (average size: 64,321 sequences/sample; min: 6,451; max: 196,691). The median Goods coverage score was ≥ 99.6% at the rarefaction point of 6,451. The software package Explicet (v2.10.5, www.explicet.org)[37] was used to calculate rarefied Good’s coverage and alpha diversity (Shannon index) measures.

### Statistical analyses

All data was prospectively collected and managed using REDCap (Research Electronic Data Capture)[38] database hosted at the University of Colorado Denver Development and Informatics Service Center. Chi-squared tests and Wilcoxon ranked sum tests were used to compare clinical characteristics across BPD groups for categorical and continuous variables, respectively. To account for differences in sequencing depth, the relative abundance (RA) of each taxon was calculated (number of sequences for specific taxa/ total number of sequences*100). Random forests (RF) consisting of 5,000 classification trees were used to multivariately evaluate microbial taxa cross-sectionally across BPD groups [39]. A random forest is an ensemble method which fits many classification trees and is often used for high dimensional data. Multidimensional Scaling using the proximity matrix from the RF was used to visualize the separation between BPD groups based on the microbial communities. Communities across collection times within a subject were compared using Shannon Beta, a measure of beta diversity [40]. This approach extends the beta diversity measure to apply to a group of samples rather than just for pair-wise comparisons. For our example, we used the following

$$H_{\beta_{i}}=\sum_{j}\frac{c_{ij}}{c_{i++}}\sum_{k}\frac{c_{ijk}}{c_{ij+}}ln(\frac{\frac{c_{ijk}}{c_{ij+}}}{\frac{c_{i+k}}{c_{+++}}})$$ (1)

where *c*_*ijk*_ is the sequence count for subject *i*, from sample *j* and taxon *k*, the + in the subscript denotes the summation of the counts over the specified indicator.

For ease of clinical interpretation, Shannon Beta is expressed as a Hill's number which indicates the effective number of communities represented by the group of samples. This measure is dependent on the number of samples from which it was calculated, and ranges from 1 to N (in our study n = 4). A normalizing transformation was used to rescale the Hill's numbers to allow comparison across subjects with differences in the number of collected samples [41].

$$H_{ni}=\frac{H_{\beta_{i}}-1}{j_{i}-1}$$ (2)

where *j*_*i*_ is the number of samples for subject *i*.

Morisita-Horn (MH, Beta-diversity) for pairwise samples within each subject were calculated. Both MH and total bacterial load were compared across the BPD severity groups using a log-normal model and generalized estimating equations to account for repeated measures.

Individual taxa of interest were modeled over time with the age of the subject at the specimen collection as the time variable and using a generalized linear mixed model. A beta-binomial join-point model was fit with a knot placed at 10 days and included a random subject effect and group specific dispersion parameters. Placement of the knot was determined by fitting a cubic function, setting the first derivative to zero and solving. Details of this analysis are described in the online data supplement (S1 Supplementary Information). The RF was performed using the RandomForest R package, all other analyses were performed using SAS version 9.4 software (SAS Institute Inc.: Cary, NC, 2014).

## Results

### Patient demographics and timing of sample collection

There were 425 infants enrolled in the parent study. Of 333 infants requiring mechanical ventilation 201 had at least one tracheal aspirate collected, and 155 of these had at least one specimen with adequate bacterial load to perform PCR for microbiome analysis (Fig 1). Characteristics for the 46 subjects with samples that could not be amplified are described in S1 Table. The median (range) age of enrollment was 3 (0–8) days. Only 3 of these subjects did not develop BPD, therefore they were excluded from further analysis due to insufficient numbers for comparison. The characteristics of the study cohort are listed in Table 1.

**Table 1:** Subject Characteristics (n = 152).

	BPD Severity					
	Mild BPD	Moderate BPD		Severe BPD		
	(n = 51)	(n = 49)		(n = 52)		
	n (%)|Mean (SD)	n (%)|Mean (SD)		n (%)|Mean (SD)		p-value
Birth Weight (g)	852.8 (159.5)	804.39 (179.5)		754.46 (132.78)		0.01
Birth Weight Z-Score	0.12 (0.6)	-0.13 (0.64)		-0.18 (0.69)		0.04
Gestational Age	25.69 (1.3)	25.61 (1.63)		25.4 (1.56)		0.61
Small for Gestational Age (SGA)	6 (12%)	9 (18%)		15 (30%)		0.89
Gender (Male)	21 (41%)	24 (49%)		28 (54%)		0.43
Maternal Ethnicity						
Hispanic or Latino	18 (35%)	18 (37%)		12 (23%)		0.26
Not Hispanic or Latino	33 (65%)	31 (63%)		40 (77%)		0.26
Maternal Complications						
Premature Rupture of Membranes	21 (41%)	17 (35%)		20 (38%)		0.80
Chorioamnionitis	11 (22%)	6 (12%)		9 (17%)		0.38
Preeclampsia	10 (20%)	13 (27%)		10 (19%)		0.70
Cesarean Section	33 (65%)	36 (73%)		35 (67%)		0.63
Days MV	18 (12)	26 (17)		56 (54)		<0.01
Pneumonia	7 (14%)	13 (27%)		15 (29%)		0.15
Surfactant	49 (96%)	47 (96%)		50 (96%)		0.99
Antenatal Corticosteroids	38 (75%)	34 (69%)		45 (87%)		0.08
Multiple gestation	7 (14%)	13 (27%)		14 (27%)		0.19

### Cross-sectional comparison of microbiome across BPD severity

**Fig 2:**
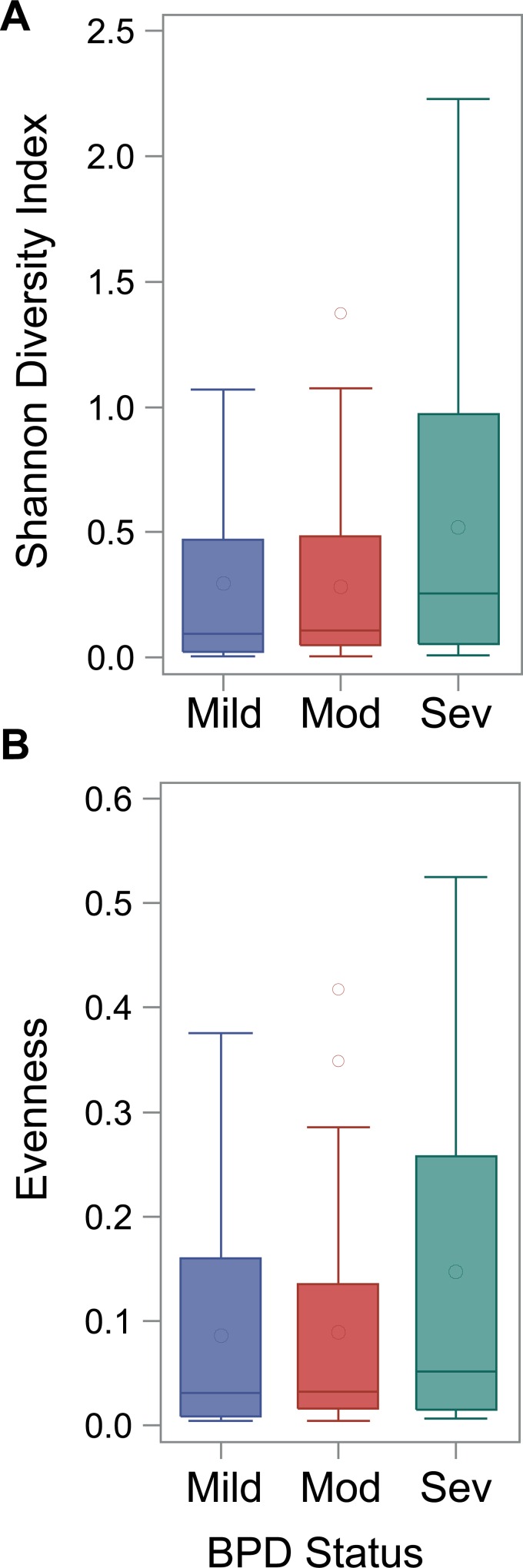
Distribution of the Shannon Diversity Index (top) and Evenness, a measure of dominance, (bottom) across BPD severity groups in cross-sectional samples collected between 5–9 days of life.

**Fig 3:**
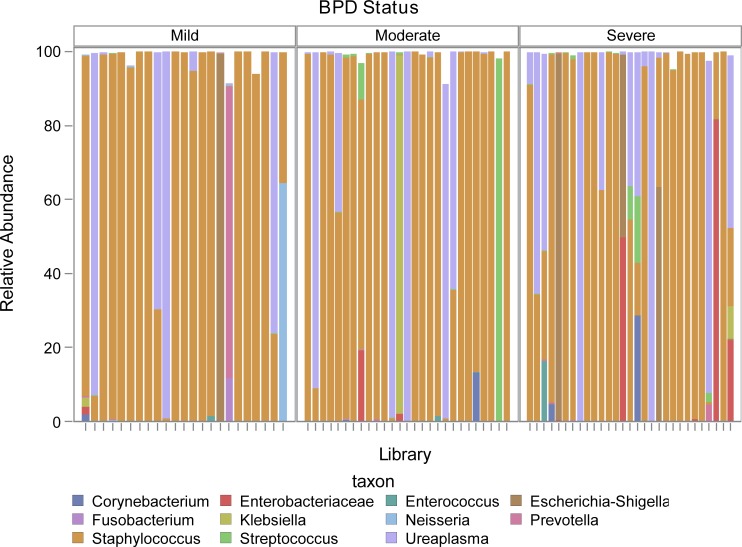
The relative abundance of bacterial communities for 7 day tracheal aspirates are displayed by BPD severity groups. Each bar represents the community of a single subject, the different taxa are represented by different colors.

The cross-sectional dataset included only samples collected between 5–9 days of age and consisted of 79 subjects: 23 mild, 27 moderate and 29 severe BPD (S2 Table). The median total bacterial load for day 7 tracheal aspirates was 4.8 log_10_ copies/reaction and ranged from 3.6 to 7.5. There was not a significant difference across BPD severity groups (p = 0.63). Shannon alpha diversity index and evenness, a measure which is small when the community is dominated by a small number of organisms, were not significantly different across BPD groups (p = 0.39 for both), although the median Shannon alpha diversity and evenness were higher in the severe BPD group (Fig 2). The bacterial communities for each subject by BPD group are displayed in Fig 3. The majority of samples were dominated by *Staphylococcus* (68%) and *Ureaplasma* (18%). The relative abundance of *Staphylococcus* was not significantly different across BPD severity (p = 0.47).

**Fig 4:**
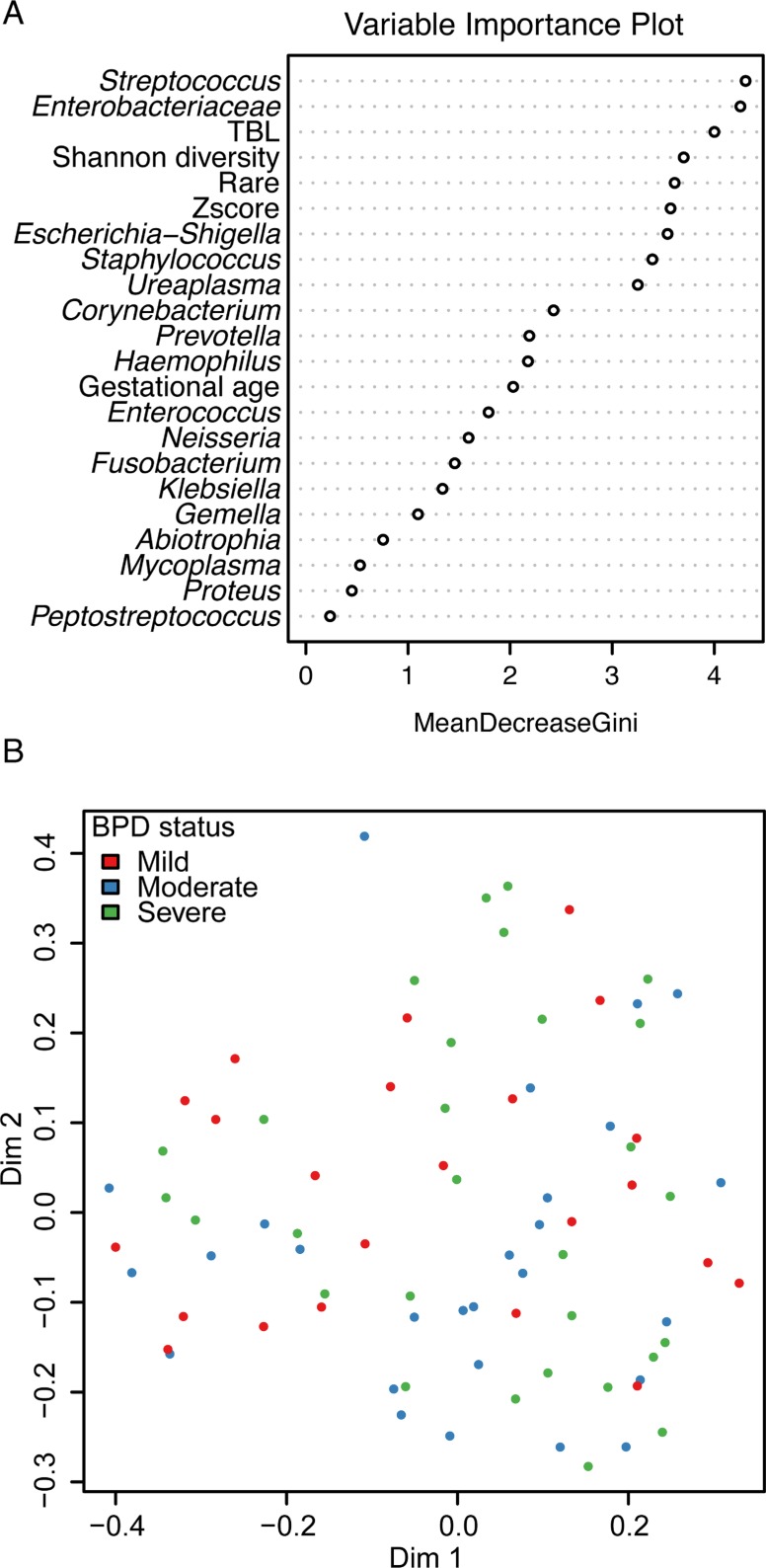
Random forests (RF) consisting of 5,000 classification trees were used to multivariately evaluate microbial taxa cross-sectionally across BPD severity groups at 7 days of age. A variable importance plot is useful for visualizing the rank importance of variables. Mean Decrease Gini Index identifies features that are ranked as more important for distinguishing BPD severity. Features with a larger value indicate variables that contribute more information compared with random noise; the separation in the indices between predictor variables degrades as the features become less useful for distinguishing between groups (top). Multidimensional Scaling plot using the proximity matrix from the RF of the 7 day tracheal aspirate specimens shows very little separation between the BPD severity groups which corresponds with the high error rate (bottom).

Microbial markers, alpha diversity, total bacterial load and the relative abundance of individual taxa (n = 18 including a rare category–criteria for rare was < 1% relative abundance in all samples) were entered as predictors in the RF to identify variables which best distinguished between the 3 BPD severity groups. The resulting RF produced an error rate of 74.7% which indicates poor association between community composition and BPD severity (Fig 4). It correctly classified 3 (13%) of the mild, 9 (33%) of the moderate and 8 (28%) of the severe BPD infants. The RF did not produce good separation between BPD severity groups based on the microbial markers. As a sensitivity analysis, clinical characteristics from Table 1 were also included as predictors in the RF, but this did not improve the error rate (76%; S1 Fig). Including a reduced subset of the 9 top predictors also did not improve the error rate (74.7%).

### Change in bacterial communities over time across BPD severity

**Fig 5:**
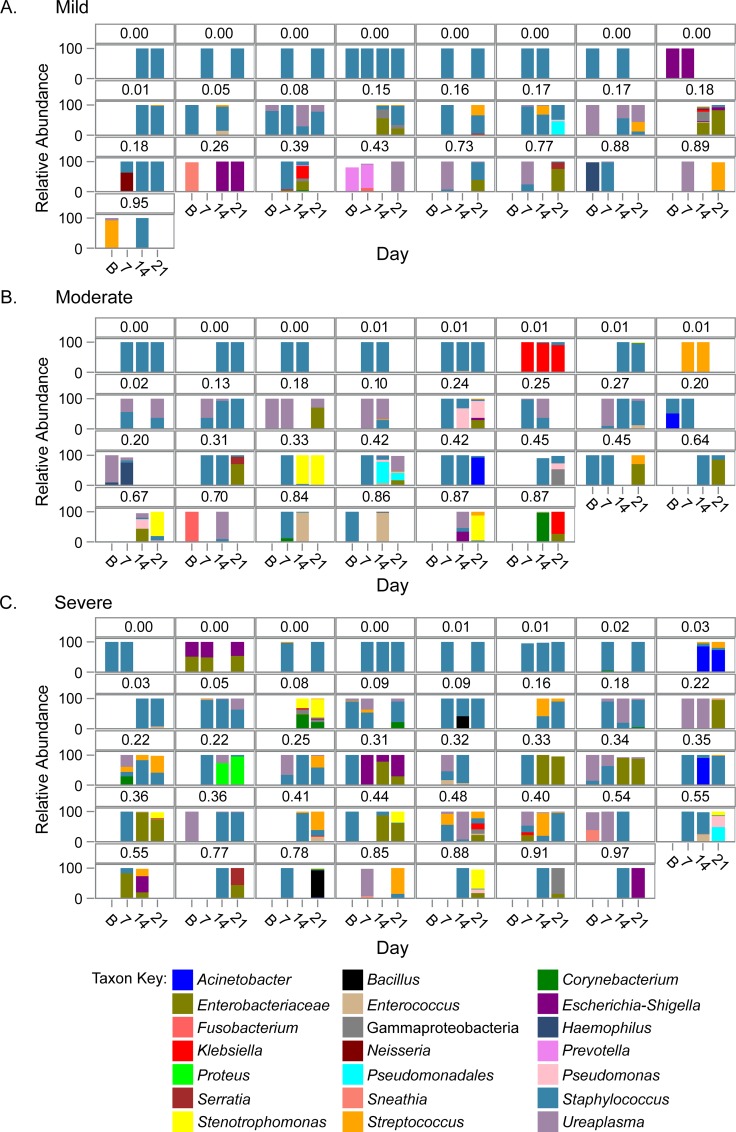
Longitudinal airway microbiome composition of each individual subject are displayed by day of collection. Samples were collected from mechanically ventilated infants at enrollment (within the first week after birth) and within 48 hours of target days 7, 14, and 21.

In addition to evaluating microbial communities cross-sectionally, we also evaluated changes in the microbial communities over time. This analysis included 233 samples that were sequenced from 94 subjects (25 mild, 30 moderate and 39 severe BPD; S3 Table). The median number of samples from a subject was 2 and ranged from 2 to 4. Total bacterial load increased on average over time by 1.05 log_10_ copies/reaction for each additional week after birth (p < 0.01). Bacterial communities from longitudinal samples collected from each subject are displayed in Fig 5, samples from the 3 subjects with no BPD are in S2 Fig.

**Fig 6:**
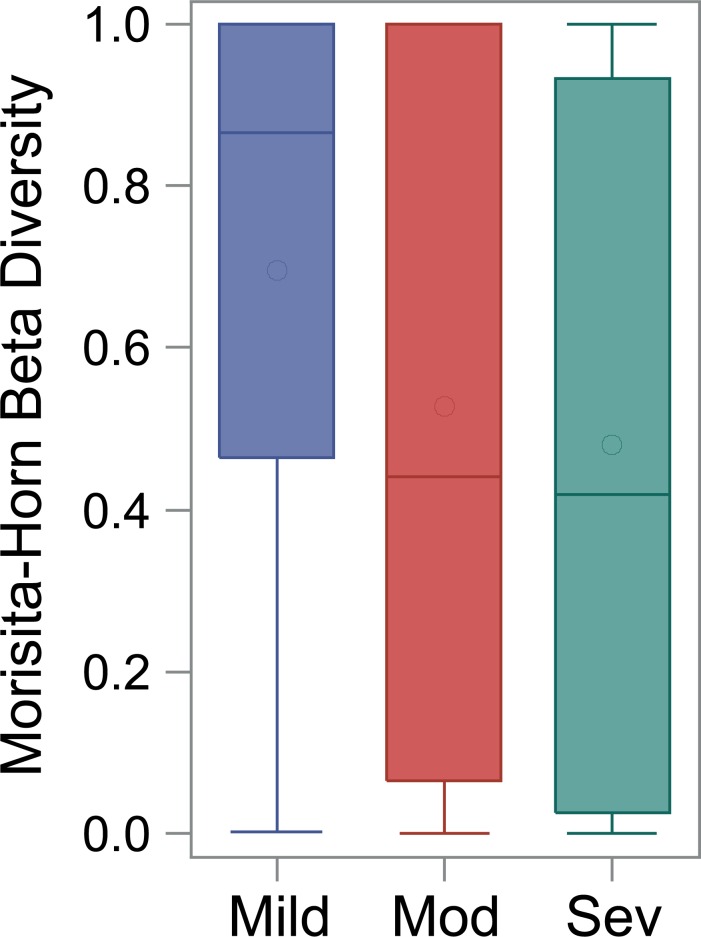
Box plots of Morisita Horn (MH) values from subjects with at least 2 samples versus disease severity. MH values were significantly lower in severe BPD compared to mild (p = 0.01) and were only marginally different between mild and moderate (p = 0.08).

A single Beta diversity measure, Shannon Beta diversity, was calculated for each subject to quantify the number of bacterial communities represented by the multiple samples. The median Beta-diversity value (higher values indicate there are more communities observed for a subject) after normalization was 0.16, 0.24 and 0.31 in the mild, moderate and severe BPD groups, respectively (ranges across groups were similar: 0 to 0.97). In addition to the single Beta diversity measure, MH, a pair-wise beta diversity measure, was calculated between pairs of samples within each subject. The mean (SE after adjusting for repeated measures) for MH values (lower values indicate increased microbial turnover) across the three severity groups were 0.69 (0.58) for mild, 0.53 (0.41) for moderate and 0.48 (0.39) for severe. MH values were significantly lower in severe BPD compared to mild (p = 0.01) and were only marginally different between mild and moderate (p = 0.08; Fig 6). S3 Fig displays the MH values by the interval days between specimen collection and indicates a trend towards greater community turnover in the moderate and severe BPD groups (p = 0.04 across all 3 groups; p = 0.05 mild vs. severe BPD). Both measures of beta diversity indicate increasing community turnover with increasing BPD severity.

**Fig 7:**
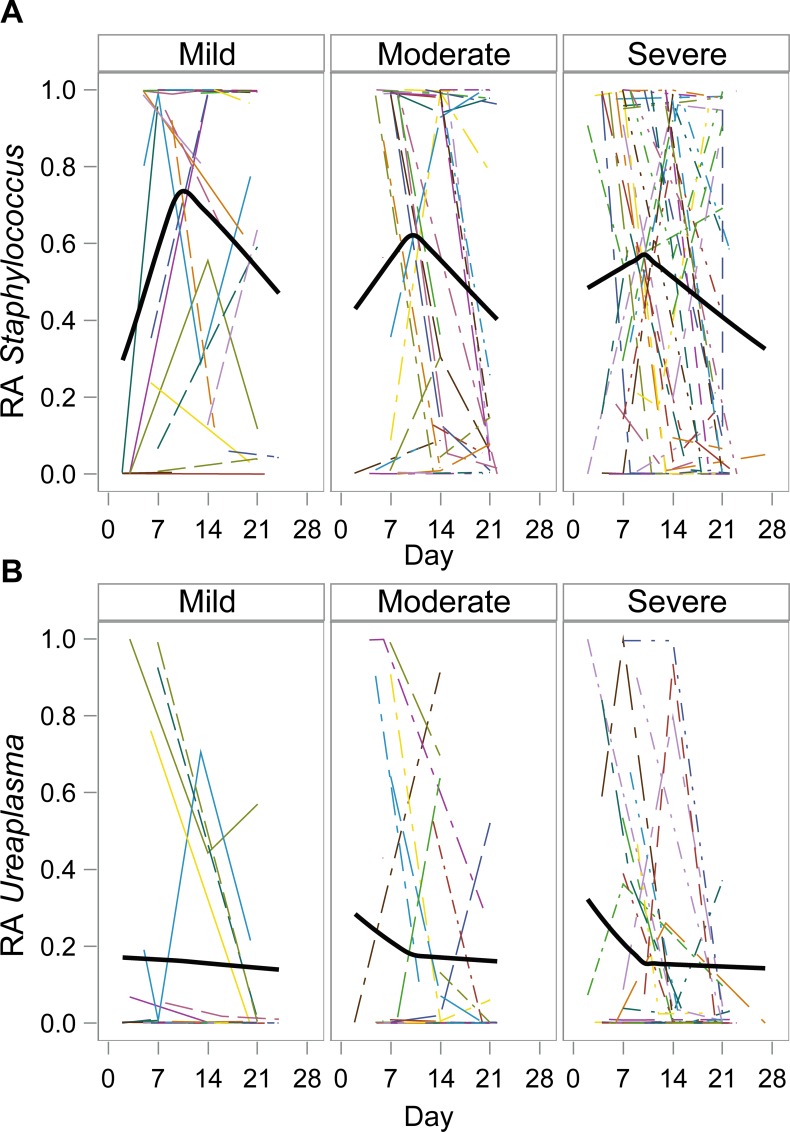
Model of Relative Abundance (RA) of *Staphylococcus* (top) and *Ureaplasma* (bottom) over time by BPD severity. The thinner lines correspond to subject specific values over time, and the thick lines denote the average trend based on the estimated parameters from the model.

Given the dominance of *Staphylococcus* and *Ureaplasma* in many of the samples, we modeled these genera over time to determine if there were consistent trends in relative abundance (RA) of these organisms. For *Staphylococcus*, the mean RA value at 14 days was higher among infants who went on to develop mild BPD compared to those who went on to develop severe BPD (p = 0.04). Fig 7 reveals that there are differences in the trends of RA over time between BPD severity groups. These data demonstrate that the RA of *Staphylococcus* in the lower airways increases at a greater rate after birth and reaches a higher absolute RA for infants who develop less severe BPD, although these trends were not statistically significant (Fig 7, top). Infants with higher RA *Ureaplasma* soon after birth were more likely to develop severe BPD, although this trend was also not statistically significant (Fig 7, bottom). For both organisms, there was more variability in the severe and moderate BPD groups compared to the mild group, which is consistent with the results from the beta diversity measures that similarly indicated an association between community changes and BPD severity. As a sensitivity analysis, the models were refit including time on ventilator and use of corticosteroids as covariates, the results remained unchanged (S1 Supplementary Information). The individual level data is not well represented by the average trend, providing support for the need of the more complex models in which individual subjects are allowed to deviate from the average.

## Discussion

We evaluated the lower airway microbiome in preterm infants requiring mechanical ventilation in both cross-sectional and longitudinal analyses to determine if there is a relationship between the early respiratory tract microbiome and BPD severity. In a cross-sectional evaluation at 7 days of age there was no evidence that bacterial communities were associated with later BPD severity. Bacterial load and Shannon diversity of airway communities did not reveal a striking pattern that was strongly linked to BPD severity either on its own or in conjunction with clinical factors. Investigation of airway microbial communities over time by several measures indicated that there may be temporal trends that are associated with BPD severity. Although, a particular organism may not necessarily be associated with BPD severity, shifts in the community of airway organisms were observed more often in preterm infants who eventually developed severe BPD. Specifically, infants who developed more severe BPD exhibited greater bacterial community turnover with increasing time from birth, acquired less *Staphylococcus* in the first days after birth, and had higher initial RA of *Ureaplasma*.

These findings are important because they reveal that the patterns and succession of the airway microbiome in preterm infants could be a marker that is predictive of BPD severity. While these data do not provide evidence that the airway microbiome directly contributes to BPD, it certainly suggests that this possibility is worthy of further investigation and could increase our understanding of the mechanisms of BPD. A predictive biomarker in this population is of enormous benefit for families eager for prognostic information, and would help guide early preventive and novel treatment strategies. Identification of microbiome patterns that are associated with outcome will also lead to clinical trials evaluating interventions (e.g. probiotics, judicious use of antibiotics, routes and content of nutritional support) that promote beneficial bacterial colonization and prevent acquisition of pathogenic bacteria, ultimately preventing or mitigating chronic lung disease of prematurity.

There have been limited evaluations of the airway microbiome in preterm infants [10, 11]. Our previous study in this area was an observation of only 10 infants, all of whom were included in the present study [10], and the study by Lohman *et al*. was performed in 25 infants [11]. This study represents a much larger cohort of 152 preterm infants from multiple hospitals, which increases the generalizability of the findings. The previous studies, along with the present one reveal that the airways of preterm infants may not be sterile at birth and quickly acquire bacteria communities that are often dominated by a single organism. Bacteria in the airways of these infants may be acquired from the mother or through exposure to the local environment in the NICU. Unfortunately, this study was not designed to evaluate the differential impact of these routes of acquisition as most infants had their first samples collected several days after birth. The study by Lohmann *et*. *al*. found that *Acinetobacter* was the frequent dominant organism in their institution [11], while our study performed at multiple hospitals affiliated with two academic centers were dominated by *Staphylococcus* and *Ureaplasma*. These differences suggest that the bacteria that populate the airways of infants may be associated with the bacterial communities in the hospital units themselves and warrants further investigation. Yet the range of different bacteria that colonize the airways of mechanically ventilated infants suggest that simple detection of specific bacteria will not be a reliable biomarker for BPD and the evaluation of entire microbial community is likely needed.

The role of *Ureaplasma* in the development of BPD remains controversial [42–46]. The finding that infants with more severe BPD tend to have higher RA of *Ureaplasma* early in their course perpetuates the possibility of its contribution to BPD [42]. Chronic intrauterine *Ureaplasma* exposure appears to downregulate the host response to acute LPS exposure in the preterm sheep model [47]. Yet, the presence of *Ureaplasma* may be a function of prematurity as Kemp et al found that host bactericidal activity was directly correlated to gestational age, and the bactericidal activity appeared related to functional complement activity [48].

Whether the rate of bacterial community turnover is directly related to BPD severity is an intriguing question. Certainly, the treatment variation among preterm infants, especially related to antibiotic administration could lead to these findings. It is possible that the increased turnover in the infants who went on to develop more severe BPD were more often treated with antibiotics because their clinical course was more severe and there were more frequent episodes of suspected infection. Future studies will have to evaluate the impact of antibiotic administration on the respiratory tract microbiome and the potential association with the development and severity of BPD.

Ecological shifts in the microbiome have been linked with increased inflammation in other human systems. Specific patterns in the acquisition and composition of the microbiome are associated with inflammation and altered epithelial barrier function in the intestinal tract of adults and premature infants [49–51]. Inflammation has been a key driver in the pathophysiology of BPD and thought to result from a variety of etiologies, including pre- and postnatal infection, mechanical ventilation, and oxygen toxicity. However, as the immune system of preterm infants is still maturing, simple colonization with bacteria may be enough to drive an inflammatory response that disrupts lung development and lead to BPD. Thus, further studies should thoroughly evaluate the interaction between the airway microbiome, the host immune response, and clinical treatment including the use of antibiotics to impact lung development in preterm infants.

There are several limitations to this study. First, because analysis of the airway microbiome requires access to the lower airway with an endotracheal tube, we were not able to assess the airway microbiome in infants who did not require mechanical ventilation. As such, we did not have sufficient numbers of infants who were mechanically ventilated that did not go on to develop BPD. The lack of a control group did not allow us to determine whether the airway microbiome could differentiate between those with and without BPD. Additionally, since fewer preterm infants are ventilated after birth due to advances in the use of non-invasive ventilation techniques and less invasive surfactant administration, the results of this study should be confined only to infants who require invasive mechanical ventilation support. Furthermore, we did not have data on antibiotic administration in this cohort and therefore were not able to evaluate its potential impact on the airway microbiome. Finally, these data were generated from a secondary analysis of a study cohort that was not specifically designed to address these questions. Therefore, these results should be interpreted as hypothesis generating, and prospective studies designed to assess the relative roles of the entire microbiome (bacteria, viruses, and fungi) are warranted.

As molecular detection of bacterial communities utilizing bacterial 16S ribosomal RNA gene sequencing has gained momentum to study the impact of the microbiome in health and disease, it has become increasingly evident that variation in the methodology employed can lead to variation in results. Hiergeist et. al. brought attention to this issue by demonstrating the variability of bacterial community detection among 9 different centers employing varying DNA extraction and 16S rRNA sequencing techniques to the same specimens [52]. While they did find that repeatedly performing the analysis with the same technique within centers produced reliable results, they did expectedly find that variation in technique across centers resulted in substantial differences in bacterial detection. Most of the variation seemed to be explained by differences in covered hypervariable (V-) regions and primers selected for 16S rDNA amplicon sequencing. Thus, careful attention to the methods utilized for molecular detection of bacteria is necessary when attempting to reproduce results from laboratory to laboratory. In this study, we employed a standard sample collection protocol across sites, and sample processing, sequencing and bioinformatics was performed at a single lab utilizing consistent and well vetted approaches. In addition, applying the same techniques to cross-sectional and longitudinal specimens as was performed in this study is sufficient to determine whether there are differences in bacterial composition across specimens collected from different subjects at the same time point as well as differences within individual subjects over time. However, determining the precise effect size of these differences is more difficult due to variation in technique.

In conclusion, longitudinal changes in airway microbial communities over time in mechanically ventilated preterm infants may be associated with BPD severity. Further evaluation is necessary to determine whether these changes are causal or in response to a change in treatment or in other factors that lead to BPD.

## Supporting Information

S1 Supplementary Information Beta-binomial parameter estimates. (DOCX)

S1 Table Subject Characteristics for the subjects with samples that did not amplify (n = 46). (DOCX)

S2 Table Subject Characteristics for the Cross-sectional Cohort (n = 79). (DOCX)

S3 Table Subject Characteristics for the Longitudinal Cohort (n = 94). (DOCX)

S1 Fig Random forests (RF) consisting of 5,000 classification trees were used to multivariately evaluate microbial taxa and the clinical data (available prior to 7 days of age) cross-sectionally across BPD groups at 7 days of age (left). Multidimensional Scaling plot using the proximity matrix from the RF shows very little separation between the BPD groups which corresponds with the high error rate (right).
(PDF)

S2 Fig Microbial composition for 3 subjects with a no BPD diagnosis. (PDF)

S3 Fig Morisita-Horn diversity values from subjects with at least 2 or more samples versus disease severity. There is a trend towards greater community turnover in the moderate and severe BPD groups (Mild n = 25, Moderate N = 39 and Severe N = 30). The bold blue line indicates the average trend.
(PDF)
