# Supplementary material for: Airway Microbial Community Turnover Differs by BPD Severity in Ventilated Preterm Infants
Source: PLoS One. 2017 Jan 27;12(1):e0170120. doi: 10.1371/journal.pone.0170120 (PMC5271346; doi:10.1371/journal.pone.0170120)
Supplement: S1 Supplementary Information — (DOCX) [file pone.0170120.s001.docx]

**Airway Microbial Community Turnover Differs by BPD Severity in Ventilated Preterm Infants**

**S1 Supplementary Information**

Brandie D. Wagner,^1,3^ Marci K. Sontag,^2^ J. Kirk Harris,^3^ Joshua I. Miller,^2^ Lindsey Morrow^1^, Charles E. Robertson,^4^ Mark Stephens,^3^ Brenda B. Poindexter,^5^ Steven H. Abman,^3,7^ and Peter M. Mourani,^6,7*^

^1^ Department of Biostatistics, Colorado School of Public Health, University of Colorado, Aurora, Colorado, United States of America

^2^ Department of Epidemiology, Colorado School of Public Health, University of Colorado Denver, Aurora, Colorado, United States of America

^3^ Section of Pulmonary, Department of Pediatrics, School of Medicine, University of Colorado, Aurora, Colorado, United States of America

^4^ Section of Infectious Disease, Department of Medicine, School of Medicine, University of Colorado, Aurora, Colorado, United States of America

^5^ Perinatal Institute, Cincinnati Children’s Hospital Medical Center, Cincinnati, Ohio, United States of America

^6^ Section of Critical Care Medicine, Department of Pediatrics, School of Medicine, University of Colorado, Aurora, Colorado, United States of America

^7^ The Pediatric Heart-Lung Center, Department of Pediatrics, School of Medicine, University of Colorado, Aurora, Colorado, United States of America

* Corresponding author

Email: peter.mourani@childrenscolorado.org (PMM)

**Methods**

*Beta-Binomial model for Microbiota data.* Microbiota data is binomially distributed as it consists of restricted count data. However, this approach makes strong assumptions about the relationship between the mean and variance of the data which have been shown to be overdispersed. There are several ways to address overdispersion which include the estimation of a scale parameter, a random effect or the use of a more general distribution. We chose to use the latter approach to allow the ability to test the amount of dispersion and to allow the expansion of the models to include random effects for our more complex study design.

In a standard binomial distribution, the binomial probability *p* is assumed to be fixed for successive trials. However, to account for overdispersion, we can assume that *p* comes from a beta distribution. Using this beta-binomial mixture distribution, the value of *p* changes for each subject, and is modeled as a random variable from a beta-distribution with shape parameters α > 0 and β > 0.

*f* (p | α, β) = $\frac{p^{\alpha-1}(1-p)^{\beta-1}}{B(\alpha,\beta)}$

where *B* is the beta function. The marginal distribution of *x* = number of sequences, is then the beta-binomial distribution,

*f* (*x* | α, β, n) = $\left(\begin{matrix} n \\ x \end{matrix}\right)$ $\frac{B(\alpha+x,n+\beta-x)}{B(\alpha,\beta)}$

Alternatively, we can reparametrize the model such that we can estimate overdispersion with parameter ρ, where $M=\alpha+\beta$ and $\rho=\frac{1}{\alpha+\beta+1}$

*Description of join-point models:* A beta-binomial join-point model was fit with a knot placed at 10 days and included a random subject effect and group specific dispersion parameters. Placement of the knot was determined by fitting a cubic function, setting the first derivative to zero and solving for days.

Using both visual inspection with a scatterplot smoother and taking the derivative of a 3^rd^ degree polynomial, a knot of 10 days was chosen.


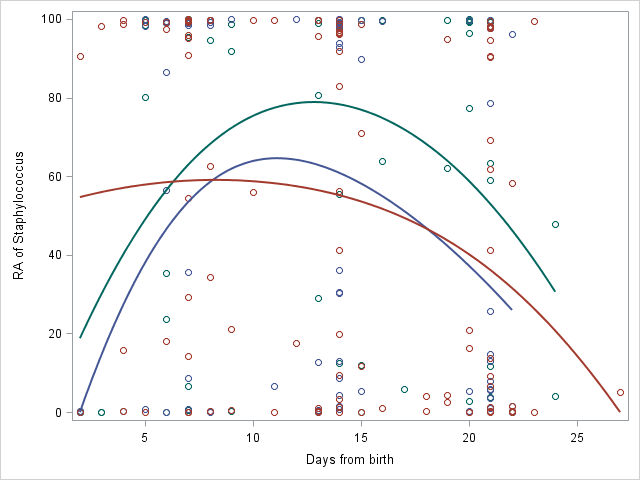


Cubic function parameters:

| **Parameter** |  | **Estimate** | **Standard Error** | **95% Confidence Limits** | | **Z** | **Pr > \|Z\|** |
| --- | --- | --- | --- | --- | --- | --- | --- |
| **bpd1** | mild | -8.8944 | 0.6522 | -10.1727 | -7.6160 | -13.64 | <.0001 |
| **bpd1** | mod | -5.1445 | 1.5892 | -8.2593 | -2.0298 | -3.24 | 0.0012 |
| **bpd1** | sev | -0.2492 | 0.6374 | -1.4985 | 1.0001 | -0.39 | 0.6958 |
| **days*bpd1** | mild | 2.2845 | 0.1673 | 1.9565 | 2.6125 | 13.65 | <.0001 |
| **days*bpd1** | mod | 1.2771 | 0.4338 | 0.4270 | 2.1273 | 2.94 | 0.0032 |
| **days*bpd1** | sev | -0.0902 | 0.1625 | -0.4088 | 0.2283 | -0.56 | 0.5788 |
| **days*days*bpd1** | mild | -0.1728 | 0.0149 | -0.2021 | -0.1436 | -11.58 | <.0001 |
| **days*days*bpd1** | mod | -0.1012 | 0.0359 | -0.1716 | -0.0309 | -2.82 | 0.0048 |
| **days*days*bpd1** | sev | 0.0082 | 0.0126 | -0.0165 | 0.0328 | 0.65 | 0.5157 |
| **days*days*days*bpd1** | mild | 0.0039 | 0.0004 | 0.0032 | 0.0047 | 9.96 | <.0001 |
| **days*days*days*bpd1** | mod | 0.0024 | 0.0009 | 0.0006 | 0.0041 | 2.64 | 0.0084 |
| **days*days*days*bpd1** | sev | -0.0003 | 0.0003 | -0.0009 | 0.0003 | -0.84 | 0.3981 |

| BPD status | d/dx | Day at which d/dx=0 |
| --- | --- | --- |
| Mild | 2.28-0.34x+0.01x^2^ | 9 |
| Moderate | 1.28-0.20x+0.0072 x^2^ | 10 |
| Severe | -0.09+0.16x-0.0009 x^2^ | 9 |

A beta-binomial join-point model was fit with a knot placed at 10 days and included a random subject effect and group specific dispersion parameters.

Logit^-1^(p_ijk_)= β_0jk_ + β_1jk_(Day_i_) + β_2jk_(Day_i_[10]) + b_i_,

ρ = Σc_k_ and M = (1-ρ)/ρ

where i = subject; j = day of sample collection, k = BPD status (2 – Mild; 3 – Moderate;

4 – Severe), Day[c] = max (Day - c, 0) and b_i_ ~ N (0, σ^2^_1_).

**SAS Code**

The SAS code below displays example NLMIXED statements for the join-point beta-binomial model with heteroscedastic regression models for Ureaplasma but could be modified for any taxa.

/*join point model*/

**proc** **nlmixed** data=model2 tech=quanew;*tech=newrap;

parms b0=-**1** b1=**.9** b2=**1.3** b3=**0.2** b4=-**0.15** b5=-**.2** b6=-**.3** b7=**.2** b8=**.2** s1u=**.2** c0=**.6** c1=**0** c2=**0**;

lp =b0 + b1*mod+ b2*sev + b3*days + b4*days*mod + b5*days*sev +

b6*day10 + b7*day10*mod + b8*day10*sev + u1;

p = exp( lp ) / (**1**+exp( lp ));

rho=(c0 + c1*mod + c2*sev);

M=(**1**-rho)/rho;

loglike=(lgamma(n + **1**)-lgamma(r + **1**)-lgamma(n - r + **1**)) + lgamma( M ) - lgamma( M*p ) - lgamma( M*(**1**-p) ) + lgamma( r + M*p ) + lgamma( n - r + M*(**1**-p) ) - lgamma( n + M );

model r ~ general (loglike);

random u1 ~ NORMAL(**0**, s1u*s1u) subject=study_id;

contrast 'int by group' b1, b2;

contrast 'slope by group' b4, b5;

contrast 'slope10 by group' b7, b8;

estimate 'mild 7days' b0 + **7***b3;

estimate 'mod 7 days' b0 + b1 + **7***b3 + **7***b4;

estimate 'sev 7days' b0 + b2 + **7***b3 + **7***b5;

estimate 'mild 10days' b0 + **10***b3;

estimate 'mod 10 days' b0 + b1 + **10***b3 + **10***b4;

estimate 'sev 10 days' b0 + b2 + **10***b3 + **10***b5;

estimate 'mild 14days' b0 + **14***b3 +**4***b6;

estimate 'mod 14 days' b0 + b1 + **14***b3 + **14***b4 + **4***b6 + **4***b7;

estimate 'sev 14days' b0 + b2 + **14***b3 + **14***b5 + **4***b6 + **4***b8;

estimate 'mild 21days' b0 + **21***b3 +**11***b6;

estimate 'mod 21 days' b0 + b1 + **21***b3 + **21***b4 + **11***b6 + **11***b7;

estimate 'sev 21days' b0 + b2 + **21***b3 + **21***b5 + **11***b6 + **11***b8;

estimate 'diff at 7 days sev vs mild' b2 + **7***b5;

estimate 'diff at 10 days sev vs mild' b2 + **10***b5;

estimate 'diff at 14 days sev vs mild' b2 + **14***b5 + **4***b8;

estimate 'diff at 21 days sev vs mild' b2 + **21***b5 + **11***b8;

contrast 'diff at 21 days sev, mod vs mild' b2 + **21***b5 + **11***b8, b1 + **21***b4 + **11***b7;

contrast 'diff at 14 days sev, mod vs mild' b2 + **14***b5 + **4***b8, b1 + **14***b4 + **4***b7;

contrast 'diff at 10 days sev, mod vs mild' b2 + **10***b5, b1 + **10***b4;

contrast 'diff at 7 days sev, mod vs mild' b2 + **7***b5, b1 + **7***b4;

predict exp(b0 + b1*mod+ b2*sev + b3*days + b4*days*mod + b5*days*sev +

b6*day10 + b7*day10*mod + b8*day10*sev)/ (**1**+ exp(b0 + b1*mod+ b2*sev

+ b3*days + b4*days*mod + b5*days*sev + b6*day10 + b7*day10*mod + b8*day10*sev)) out=pred2;

**run**;

Model Parameters estimated for Staphylococcus

| **Parameter** | **Estimate** | **Standard Error** | **DF** | **t Value** | **Pr > \|t\|** | **Lower** | **Upper** |
| --- | --- | --- | --- | --- | --- | --- | --- |
| **b0** | -1.3532 | 0.6128 | 93 | -2.21 | 0.0297 | -2.5701 | -0.1362 |
| **b1** | 0.8640 | 1.0528 | 93 | 0.82 | 0.4139 | -1.2266 | 2.9546 |
| **b2** | 1.2023 | 0.9029 | 93 | 1.33 | 0.1863 | -0.5907 | 2.9952 |
| **b3** | 0.2442 | 0.09258 | 93 | 2.64 | 0.0098 | 0.06039 | 0.4281 |
| **b4** | -0.1409 | 0.1447 | 93 | -0.97 | 0.3327 | -0.4282 | 0.1464 |
| **b5** | -0.2006 | 0.1254 | 93 | -1.60 | 0.1132 | -0.4496 | 0.04849 |
| **b6** | -0.3305 | 0.1352 | 93 | -2.44 | 0.0164 | -0.5990 | -0.06193 |
| **b7** | 0.1488 | 0.2011 | 93 | 0.74 | 0.4614 | -0.2507 | 0.5482 |
| **b8** | 0.2271 | 0.1747 | 93 | 1.30 | 0.1968 | -0.1198 | 0.5741 |
| **s1u** | 0.2284 | 0.2828 | 93 | 0.81 | 0.4213 | -0.3331 | 0.7900 |
| **c0** | 0.6190 | 0.04077 | 93 | 15.18 | <.0001 | 0.5380 | 0.7000 |
| **c1** | 0.02130 | 0.04644 | 93 | 0.46 | 0.6475 | -0.07091 | 0.1135 |
| **c2** | 0.01467 | 0.04377 | 93 | 0.34 | 0.7384 | -0.07226 | 0.1016 |

| **Contrasts** | | | | |
| --- | --- | --- | --- | --- |
| **Label** | **Num DF** | **Den DF** | **F Value** | **Pr > F** |
| **int by group** | 2 | 93 | 0.94 | 0.3938 |
| **slope by group** | 2 | 93 | 1.31 | 0.2740 |
| **slope10 by group** | 2 | 93 | 0.85 | 0.4314 |
| **diff at 21 days sev, mod vs mild** | 2 | 93 | 0.94 | 0.3953 |
| **diff at 14 days sev, mod vs mild** | 2 | 93 | 2.17 | 0.1205 |
| **diff at 10 days sev, mod vs mild** | 2 | 93 | 1.18 | 0.3109 |
| **diff at 7 days sev, mod vs mild** | 2 | 93 | 0.19 | 0.8246 |

| **Additional Estimates** | | | | | | | | |
| --- | --- | --- | --- | --- | --- | --- | --- | --- |
| **Label** | **Estimate** | **Standard Error** | **DF** | **t Value** | **Pr > \|t\|** | **Alpha** | **Lower** | **Upper** |
| **mild 7days** | 0.3565 | 0.2516 | 93 | 1.42 | 0.1598 | 0.05 | -0.1431 | 0.8562 |
| **mod 7 days** | 0.2343 | 0.2379 | 93 | 0.98 | 0.3273 | 0.05 | -0.2382 | 0.7068 |
| **sev 7days** | 0.1549 | 0.2035 | 93 | 0.76 | 0.4484 | 0.05 | -0.2491 | 0.5589 |
| **mild 10 days** | 1.0892 | 0.4320 | 93 | 2.52 | 0.0134 | 0.05 | 0.2313 | 1.9471 |
| **mod 10 days** | 0.5444 | 0.3721 | 93 | 1.46 | 0.1469 | 0.05 | -0.1946 | 1.2834 |
| **sev 10 days** | 0.2860 | 0.2921 | 93 | 0.98 | 0.3301 | 0.05 | -0.2941 | 0.8660 |
| **mild 14days** | 0.7443 | 0.2770 | 93 | 2.69 | 0.0085 | 0.05 | 0.1943 | 1.2944 |
| **mod 14 days** | 0.2311 | 0.2303 | 93 | 1.00 | 0.3184 | 0.05 | -0.2264 | 0.6885 |
| **sev 14days** | 0.04735 | 0.1894 | 93 | 0.25 | 0.8031 | 0.05 | -0.3287 | 0.4234 |
| **mild 21days** | 0.1408 | 0.3129 | 93 | 0.45 | 0.6538 | 0.05 | -0.4805 | 0.7621 |
| **mod 21 days** | -0.3173 | 0.3050 | 93 | -1.04 | 0.3009 | 0.05 | -0.9228 | 0.2883 |
| **sev 21days** | -0.3702 | 0.2228 | 93 | -1.66 | 0.0999 | 0.05 | -0.8126 | 0.07221 |
| **diff at 7 days sev vs mild** | -0.2016 | 0.3243 | 93 | -0.62 | 0.5357 | 0.05 | -0.8457 | 0.4424 |
| **diff at 10 days sev vs mild** | -0.8033 | 0.5222 | 93 | -1.54 | 0.1274 | 0.05 | -1.8404 | 0.2338 |
| **diff at 14 days sev vs mild** | -0.6970 | 0.3358 | 93 | -2.08 | 0.0407 | 0.05 | -1.3637 | -0.03023 |
| **diff at 21 days sev vs mild** | -0.5110 | 0.3843 | 93 | -1.33 | 0.1869 | 0.05 | -1.2742 | 0.2522 |

Model Parameters estimated for Staphylococcus with clinical covariates

| **Parameter** | **Estimate** | **Standard Error** | **DF** | **t Value** | **Pr > \|t\|** | **Lower** | **Upper** |
| --- | --- | --- | --- | --- | --- | --- | --- |
| **b0** | -1.5284 | 0.6650 | 93 | -2.30 | 0.0238 | -2.8489 | -0.2079 |
| **b1** | 0.8340 | 1.0575 | 93 | 0.79 | 0.4323 | -1.2660 | 2.9340 |
| **b2** | 1.1222 | 0.9126 | 93 | 1.23 | 0.2219 | -0.6900 | 2.9344 |
| **b3** | 0.2463 | 0.09292 | 93 | 2.65 | 0.0094 | 0.06182 | 0.4309 |
| **b4** | -0.1390 | 0.1451 | 93 | -0.96 | 0.3405 | -0.4272 | 0.1491 |
| **b5** | -0.2015 | 0.1256 | 93 | -1.60 | 0.1119 | -0.4509 | 0.04783 |
| **b6** | -0.3294 | 0.1351 | 93 | -2.44 | 0.0167 | -0.5977 | -0.06107 |
| **b7** | 0.1418 | 0.2015 | 93 | 0.70 | 0.4834 | -0.2584 | 0.5421 |
| **b8** | 0.2240 | 0.1748 | 93 | 1.28 | 0.2031 | -0.1231 | 0.5711 |
| **s1u** | 0.2472 | 0.2520 | 93 | 0.98 | 0.3291 | -0.2532 | 0.7476 |
| **c0** | 0.6153 | 0.04065 | 93 | 15.13 | <.0001 | 0.5345 | 0.6960 |
| **c1** | 0.02419 | 0.04707 | 93 | 0.51 | 0.6085 | -0.06928 | 0.1177 |
| **c2** | 0.01707 | 0.04418 | 93 | 0.39 | 0.7001 | -0.07066 | 0.1048 |
| **b9-ventdays** | 0.001680 | 0.002336 | 93 | 0.72 | 0.4738 | -0.00296 | 0.006320 |
| **b10-steroids** | 0.1619 | 0.2525 | 93 | 0.64 | 0.5229 | -0.3395 | 0.6633 |

| **Contrasts** | | | | |
| --- | --- | --- | --- | --- |
| **Label** | **Num DF** | **Den DF** | **F Value** | **Pr > F** |
| **int by group** | 2 | 93 | 0.81 | 0.4466 |
| **slope by group** | 2 | 93 | 1.32 | 0.2728 |
| **slope10 by group** | 2 | 93 | 0.82 | 0.4423 |
| **diff at 21 days sev, mod vs mild** | 2 | 93 | 1.28 | 0.2824 |
| **diff at 14 days sev, mod vs mild** | 2 | 93 | 2.54 | 0.0844 |
| **diff at 10 days sev, mod vs mild** | 2 | 93 | 1.41 | 0.2493 |
| **diff at 7 days sev, mod vs mild** | 2 | 93 | 0.36 | 0.6993 |

| **Label** | **Estimate** | **Standard Error** | **DF** | **t Value** | **Pr > \|t\|** | **Lower** | **Upper** |
| --- | --- | --- | --- | --- | --- | --- | --- |
| **mild 7days** | 0.1960 | 0.3222 | 93 | 0.61 | 0.5444 | -0.4438 | 0.8359 |
| **mod 7 days** | 0.05689 | 0.3223 | 93 | 0.18 | 0.8603 | -0.5832 | 0.6970 |
| **sev 7days** | -0.09241 | 0.3574 | 93 | -0.26 | 0.7965 | -0.8020 | 0.6172 |
| **mild 10 days** | 0.9351 | 0.4667 | 93 | 2.00 | 0.0480 | 0.008228 | 1.8619 |
| **mod 10 days** | 0.3789 | 0.4226 | 93 | 0.90 | 0.3723 | -0.4603 | 1.2180 |
| **sev 10 days** | 0.04207 | 0.4015 | 93 | 0.10 | 0.9168 | -0.7551 | 0.8393 |
| **mild 14days** | 0.6028 | 0.3236 | 93 | 1.86 | 0.0656 | -0.03969 | 1.2454 |
| **mod 14 days** | 0.05788 | 0.3108 | 93 | 0.19 | 0.8527 | -0.5594 | 0.6752 |
| **sev 14days** | -0.2001 | 0.3391 | 93 | -0.59 | 0.5566 | -0.8736 | 0.4734 |
| **mild 21days** | 0.02144 | 0.3450 | 93 | 0.06 | 0.9506 | -0.6638 | 0.7066 |
| **mod 21 days** | -0.5039 | 0.3805 | 93 | -1.32 | 0.1887 | -1.2594 | 0.2517 |
| **sev 21days** | -0.6239 | 0.3675 | 93 | -1.70 | 0.0929 | -1.3536 | 0.1058 |
| **diff at 7 days sev vs mild** | -0.2884 | 0.3423 | 93 | -0.84 | 0.4016 | -0.9683 | 0.3914 |
| **diff at 10 days sev vs mild** | -0.8930 | 0.5325 | 93 | -1.68 | 0.0969 | -1.9504 | 0.1643 |
| **diff at 14 days sev vs mild** | -0.8029 | 0.3568 | 93 | -2.25 | 0.0268 | -1.5115 | -0.09442 |
| **diff at 21 days sev vs mild** | -0.6453 | 0.4107 | 93 | -1.57 | 0.1195 | -1.4609 | 0.1702 |

Model Parameter estimated for Ureaplasma

| **Parameter** | **Estimate** | **Standard Error** | **DF** | **t Value** | **Pr > \|t\|** | **Lower** | **Upper** |
| --- | --- | --- | --- | --- | --- | --- | --- |
| **b0** | -1.5649 | 0.5873 | 93 | -2.66 | 0.0091 | -2.7311 | -0.3987 |
| **b1** | 0.7941 | 0.9981 | 93 | 0.80 | 0.4283 | -1.1880 | 2.7762 |
| **b2** | 1.0571 | 0.8572 | 93 | 1.23 | 0.2206 | -0.6452 | 2.7593 |
| **b3** | -0.00751 | 0.08062 | 93 | -0.09 | 0.9259 | -0.1676 | 0.1526 |
| **b4** | -0.06963 | 0.1301 | 93 | -0.54 | 0.5938 | -0.3280 | 0.1888 |
| **b5** | -0.1106 | 0.1132 | 93 | -0.98 | 0.3314 | -0.3354 | 0.1143 |
| **b6** | -0.00537 | 0.1160 | 93 | -0.05 | 0.9632 | -0.2358 | 0.2250 |
| **b7** | 0.07338 | 0.1770 | 93 | 0.41 | 0.6795 | -0.2782 | 0.4249 |
| **b8** | 0.1173 | 0.1552 | 93 | 0.76 | 0.4515 | -0.1908 | 0.4255 |
| **s1u** | 0.000827 | 0.1926 | 93 | 0.00 | 0.9966 | -0.3816 | 0.3832 |
| **c0** | 0.5405 | 0.05336 | 93 | 10.13 | <.0001 | 0.4345 | 0.6465 |
| **c1** | 0.04276 | 0.06950 | 93 | 0.62 | 0.5400 | -0.09527 | 0.1808 |
| **c2** | 0.01956 | 0.06623 | 93 | 0.30 | 0.7684 | -0.1120 | 0.1511 |

| **Contrasts** | | | | |
| --- | --- | --- | --- | --- |
| **Label** | **Num DF** | **Den DF** | **F Value** | **Pr > F** |
| **int by group** | 2 | 93 | 0.81 | 0.4464 |
| **slope by group** | 2 | 93 | 0.48 | 0.6182 |
| **slope10 by group** | 2 | 93 | 0.29 | 0.7511 |
| **diff at 21 days sev, mod vs mild** | 2 | 93 | 0.06 | 0.9409 |
| **diff at 14 days sev, mod vs mild** | 2 | 93 | 0.09 | 0.9154 |
| **diff at 10 days sev, mod vs mild** | 2 | 93 | 0.05 | 0.9509 |
| **diff at 7 days sev, mod vs mild** | 2 | 93 | 0.41 | 0.6619 |

| **Additional Estimates** | | | | | | | | |
| --- | --- | --- | --- | --- | --- | --- | --- | --- |
| **Label** | **Estimate** | **Standard Error** | **DF** | **t Value** | **Pr > \|t\|** | **Alpha** | **Lower** | **Upper** |
| **mild 7days** | -1.6175 | 0.2782 | 93 | -5.81 | <.0001 | 0.05 | -2.1699 | -1.0651 |
| **mod 7 days** | -1.3108 | 0.2537 | 93 | -5.17 | <.0001 | 0.05 | -1.8145 | -0.8071 |
| **sev 7days** | -1.3343 | 0.2143 | 93 | -6.23 | <.0001 | 0.05 | -1.7599 | -0.9087 |
| **mild 10days** | -1.6401 | 0.3972 | 93 | -4.13 | <.0001 | 0.05 | -2.4287 | -0.8514 |
| **mod 10 days** | -1.5422 | 0.3545 | 93 | -4.35 | <.0001 | 0.05 | -2.2461 | -0.8384 |
| **sev 10 days** | -1.6885 | 0.2968 | 93 | -5.69 | <.0001 | 0.05 | -2.2778 | -1.0992 |
| **mild 14days** | -1.6916 | 0.2869 | 93 | -5.90 | <.0001 | 0.05 | -2.2614 | -1.1218 |
| **mod 14 days** | -1.5788 | 0.2499 | 93 | -6.32 | <.0001 | 0.05 | -2.0751 | -1.0825 |
| **sev 14days** | -1.7129 | 0.2159 | 93 | -7.93 | <.0001 | 0.05 | -2.1416 | -1.2843 |
| **mild 21days** | -1.7818 | 0.3272 | 93 | -5.45 | <.0001 | 0.05 | -2.4315 | -1.1321 |
| **mod 21 days** | -1.6427 | 0.2996 | 93 | -5.48 | <.0001 | 0.05 | -2.2377 | -1.0477 |
| **sev 21days** | -1.7557 | 0.2340 | 93 | -7.50 | <.0001 | 0.05 | -2.2203 | -1.2911 |
| **diff at 7 days sev vs mild** | 0.2832 | 0.3511 | 93 | 0.81 | 0.4220 | 0.05 | -0.4141 | 0.9805 |
| **diff at 10 days sev vs mild** | -0.04847 | 0.4958 | 93 | -0.10 | 0.9223 | 0.05 | -1.0330 | 0.9360 |
| **diff at 14 days sev vs mild** | -0.02135 | 0.3591 | 93 | -0.06 | 0.9527 | 0.05 | -0.7344 | 0.6917 |
| **diff at 21 days sev vs mild** | 0.02610 | 0.4022 | 93 | 0.06 | 0.9484 | 0.05 | -0.7726 | 0.8248 |

Model Parameters estimated for Ureaplasma with clinical covariates

| **Parameter** | **Estimate** | **Standard Error** | **DF** | **t Value** | **Pr > \|t\|** | **Lower** | **Upper** |
| --- | --- | --- | --- | --- | --- | --- | --- |
| **b0** | -1.6506 | 0.6250 | 93 | -2.64 | 0.0097 | -2.8917 | -0.4095 |
| **b1** | 0.7669 | 0.9998 | 93 | 0.77 | 0.4450 | -1.2184 | 2.7523 |
| **b2** | 1.0100 | 0.8680 | 93 | 1.16 | 0.2475 | -0.7136 | 2.7337 |
| **b3** | -0.00774 | 0.08084 | 93 | -0.10 | 0.9240 | -0.1683 | 0.1528 |
| **b4** | -0.06639 | 0.1302 | 93 | -0.51 | 0.6115 | -0.3250 | 0.1923 |
| **b5** | -0.1079 | 0.1135 | 93 | -0.95 | 0.3443 | -0.3332 | 0.1175 |
| **b6** | -0.00207 | 0.1162 | 93 | -0.02 | 0.9858 | -0.2328 | 0.2286 |
| **b7** | 0.06652 | 0.1772 | 93 | 0.38 | 0.7082 | -0.2854 | 0.4184 |
| **b8** | 0.1108 | 0.1557 | 93 | 0.71 | 0.4785 | -0.1984 | 0.4200 |
| **s1u** | -0.00077 | 0.1921 | 93 | -0.00 | 0.9968 | -0.3822 | 0.3806 |
| **c0** | 0.5406 | 0.05336 | 93 | 10.13 | <.0001 | 0.4346 | 0.6466 |
| **c1** | 0.04288 | 0.06951 | 93 | 0.62 | 0.5388 | -0.09516 | 0.1809 |
| **c2** | 0.01909 | 0.06623 | 93 | 0.29 | 0.7738 | -0.1124 | 0.1506 |
| **b9** | 0.000334 | 0.001905 | 93 | 0.18 | 0.8611 | -0.00345 | 0.004117 |
| **b10** | 0.1026 | 0.2142 | 93 | 0.48 | 0.6331 | -0.3227 | 0.5279 |

| **Contrasts** | | | | |
| --- | --- | --- | --- | --- |
| **Label** | **Num DF** | **Den DF** | **F Value** | **Pr > F** |
| **int by group** | 2 | 93 | 0.73 | 0.4846 |
| **slope by group** | 2 | 93 | 0.46 | 0.6347 |
| **slope10 by group** | 2 | 93 | 0.25 | 0.7765 |
| **diff at 21 days sev, mod vs mild** | 2 | 93 | 0.07 | 0.9340 |
| **diff at 14 days sev, mod vs mild** | 2 | 93 | 0.12 | 0.8911 |
| **diff at 10 days sev, mod vs mild** | 2 | 93 | 0.07 | 0.9343 |
| **diff at 7 days sev, mod vs mild** | 2 | 93 | 0.36 | 0.6962 |

| **Label** | **Estimate** | **Standard Error** | **DF** | **t Value** | **Pr > \|t\|** | **Lower** | **Upper** |
| --- | --- | --- | --- | --- | --- | --- | --- |
| **mild 7days** | -1.7047 | 0.3323 | 93 | -5.13 | <.0001 | -2.3645 | -1.0449 |
| **mod 7 days** | -1.4025 | 0.3158 | 93 | -4.44 | <.0001 | -2.0297 | -0.7753 |
| **sev 7days** | -1.4499 | 0.3311 | 93 | -4.38 | <.0001 | -2.1073 | -0.7924 |
| **mild 10 days** | -1.7279 | 0.4317 | 93 | -4.00 | 0.0001 | -2.5852 | -0.8706 |
| **mod 10 days** | -1.6249 | 0.3978 | 93 | -4.09 | <.0001 | -2.4148 | -0.8350 |
| **sev 10 days** | -1.7967 | 0.3751 | 93 | -4.79 | <.0001 | -2.5416 | -1.0517 |
| **mild 14days** | -1.7672 | 0.3240 | 93 | -5.45 | <.0001 | -2.4107 | -1.1237 |
| **mod 14 days** | -1.6636 | 0.3084 | 93 | -5.39 | <.0001 | -2.2760 | -1.0511 |
| **sev 14days** | -1.8243 | 0.3208 | 93 | -5.69 | <.0001 | -2.4614 | -1.1872 |
| **mild 21days** | -1.8358 | 0.3482 | 93 | -5.27 | <.0001 | -2.5272 | -1.1444 |
| **mod 21 days** | -1.7313 | 0.3503 | 93 | -4.94 | <.0001 | -2.4269 | -1.0357 |
| **sev 21days** | -1.8726 | 0.3431 | 93 | -5.46 | <.0001 | -2.5540 | -1.1912 |
| **diff at 7 days sev vs mild** | 0.2549 | 0.3626 | 93 | 0.70 | 0.4839 | -0.4652 | 0.9749 |
| **diff at 10 days sev vs mild** | -0.06875 | 0.5014 | 93 | -0.14 | 0.8912 | -1.0643 | 0.9268 |
| **diff at 14 days sev vs mild** | -0.05711 | 0.3708 | 93 | -0.15 | 0.8779 | -0.7934 | 0.6792 |
| **diff at 21 days sev vs mild** | -0.03674 | 0.4232 | 93 | -0.09 | 0.9310 | -0.8772 | 0.8037 |
