# Supplementary material for: Airway Microbial Community Turnover Differs by BPD Severity in Ventilated Preterm Infants
Source: PLoS One. 2017 Jan 27;12(1):e0170120. doi: 10.1371/journal.pone.0170120 (PMC5271346; doi:10.1371/journal.pone.0170120)
Supplement: S1 Table — (DOCX) [file pone.0170120.s002.docx]

**S1 Table.** Subject Characteristics for the subjects with samples that did not amplify (n = 46).

| n (%)\|Mean (SD) | (n= 46) |
| --- | --- |
| Birth Weight Z-Score | -0.2 (0.7) |
| Gestational Age | 27 (2) |
| Small for Gestational age | 7 (15%) |
| Gender (Male) | 20 (43%) |
| Maternal Ethnicity |  |
| Not Hispanic or Latino | 34 (74%) |
| Maternal Complications |  |
| Premature Rupture of Membranes | 14 (30%) |
| Chorioamnionitis | 9 (20%) |
| Preeclampsia | 10 (22%) |
| Cesarean Section | 33 (72%) |
| Days MV | 9 (1 – 108) |
| Pneumonia | 1 (2%) |
| Surfactant | 45 (98%) |
| Antenatal Corticosteroids | 37 (80%) |
| Multiple gestation | 14 (30%) |
| BPD Severity |  |
| None | 8 (17%) |
| Mild | 20 (43%) |
| Moderate | 4 (9%) |
| Severe | 14 (30%) |
| Age at Aspirate sample collection | 4 (1 – 21) |
