# Supplementary material for: Airway Microbial Community Turnover Differs by BPD Severity in Ventilated Preterm Infants
Source: PLoS One. 2017 Jan 27;12(1):e0170120. doi: 10.1371/journal.pone.0170120 (PMC5271346; doi:10.1371/journal.pone.0170120)
Supplement: S2 Table — (DOCX) [file pone.0170120.s003.docx]

**S2 Table.** Subject Characteristics for the Cross-sectional Cohort (n = 79).

|  | BPD Severity | | |  |
| --- | --- | --- | --- | --- |
| n (%)\|Mean (SD) | Mild BPD  (n= 23) | Moderate BPD (n= 27) | Severe BPD (n= 29) | p-value |
| Birth Weight (g) | 803.7 (128.77) | 737.56 (150.4) | 760 (146.67) | 0.264 |
| Birth Weight Z-Score | 0.05 (0.63) | -0.16 (0.59) | -0.08 (0.58) | 0.479 |
| Gestational Age | 25.52 (1.34) | 25.11 (1.37) | 25.21 (1.59) | 0.586 |
| Small for Gestational age | 4 (16%) | 7 (23%) | 8 (21%) | 0.795 |
| Gender (Male) | 7 (30.4%) | 10 (37%) | 15 (51.7%) | 0.270 |
| Maternal Ethnicity |  |  |  |  |
| Hispanic or Latino | 9 (39.1%) | 11 (40.7%) | 10 (34.5%) | 0.882 |
| Not Hispanic or Latino | 14 (60.9%) | 16 (59.3%) | 19 (65.5%) | 0.882 |
| Maternal Complications |  |  |  |  |
| Premature Rupture of Membranes | 11 (47.8%) | 7 (25.9%) | 13 (44.8%) | 0.212 |
| Chorioamnionitis | 5 (21.7%) | 2 (7.4%) | 7 (24.1%) | 0.145 |
| Preeclampsia | 4 (17.4%) | 9 (33.3%) | 6 (20.7%) | 0.479 |
| Cesarean Section | 15 (65.2%) | 20 (74.1%) | 18 (62.1%) | 0.618 |
| Days MV | 20 (12) | 32 (19) | 69 (62) | <0.01 |
| Pneumonia | 3 (13%) | 10 (37%) | 12 (41%) | 0.07 |
| Surfactant | 22 (96%) | 26 (96%) | 29 (100%) | 0.54 |
| Antenatal Corticosteroids | 19 (83%) | 19 (70%) | 28 (97%) | 0.03 |
| Multiple gestation | 4 (17%) | 7 (26%) | 4 (14%) | 0.50 |
