# Supplementary material for: Airway Microbial Community Turnover Differs by BPD Severity in Ventilated Preterm Infants
Source: PLoS One. 2017 Jan 27;12(1):e0170120. doi: 10.1371/journal.pone.0170120 (PMC5271346; doi:10.1371/journal.pone.0170120)
Supplement: S3 Table — (DOCX) [file pone.0170120.s004.docx]

**S3 Table.** Subject Characteristics for the Longitudinal Cohort (n = 94).

|  | BPD Severity | | |  |
| --- | --- | --- | --- | --- |
| n (%)\|Mean (SD) | Mild BPD  (n= 25) | Moderate BPD  (n= 30) | Severe BPD  (n= 39) | p-value |
| Birth Weight (g) | 795 (149.19) | 727 (142.74) | 766.15 (139.28) | 0.212 |
| Birth Weight Z-Score | 0.17 (0.66) | -0.18 (0.65) | -0.07 (0.58) | 0.119 |
| Gestational Age | 25.16 (1.25) | 25 (1.2) | 25.26 (1.46) | 0.729 |
| Small for Gestational Age | 3 (13%) | 5 (19%) | 5 (17%) | 0.864 |
| Gender (Male) | 8 (32%) | 14 (46.7%) | 20 (51.3%) | 0.307 |
| Maternal Ethnicity |  |  |  |  |
| Hispanic or Latino | 10 (40%) | 12 (40%) | 10 (25.6%) | 0.351 |
| Not Hispanic or Latino | 15 (60%) | 18 (60%) | 29 (74.4%) | 0.351 |
| Maternal Complications |  |  |  |  |
| Premature Rupture of Membranes | 12 (48%) | 9 (30%) | 15 (38.5%) | 0.392 |
| Chorioamnionitis | 5 (20%) | 3 (10%) | 8 (20.5%) | 0.354 |
| Preeclampsia | 3 (12%) | 9 (30%) | 9 (23.1%) | 0.358 |
| Cesarean Section | 14 (56%) | 22 (73.3%) | 25 (64.1%) | 0.403 |
| Days MV | 21 (13) | 33 (18) | 59 (58) | <0.01 |
| Pneumonia | 5 (20%) | 12 (40%) | 13 (33%) | 0.28 |
| Surfactant | 24 (96%) | 29 (97%) | 39 (100%) | 0.48 |
| Antenatal Corticosteroids | 17 (68%) | 21 (70%) | 36 (92%) | 0.05 |
| Multiple gestation | 3 (12%) | 6 (20%) | 7 (18%) | 0.72 |
