# Supplementary figures and images for: Airway Microbial Community Turnover Differs by BPD Severity in Ventilated Preterm Infants
Source: PLoS One. 2017 Jan 27;12(1):e0170120. doi: 10.1371/journal.pone.0170120 (PMC5271346; doi:10.1371/journal.pone.0170120)

### Variable Importance Plot

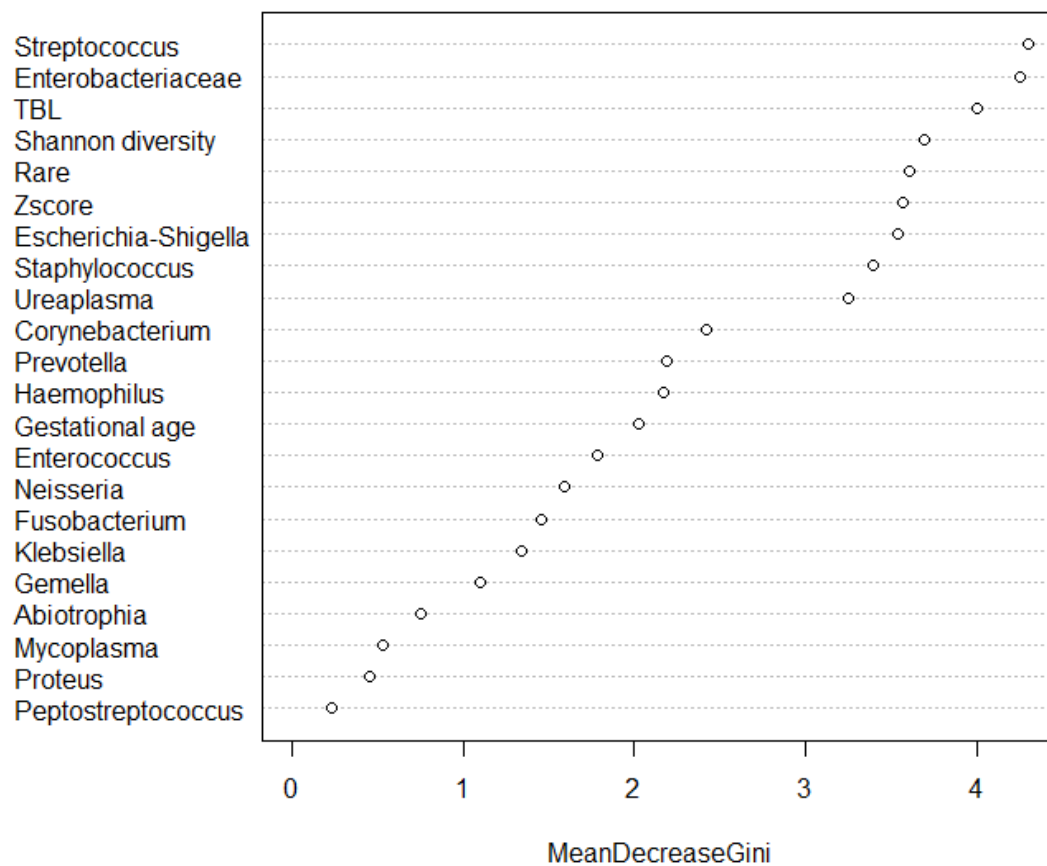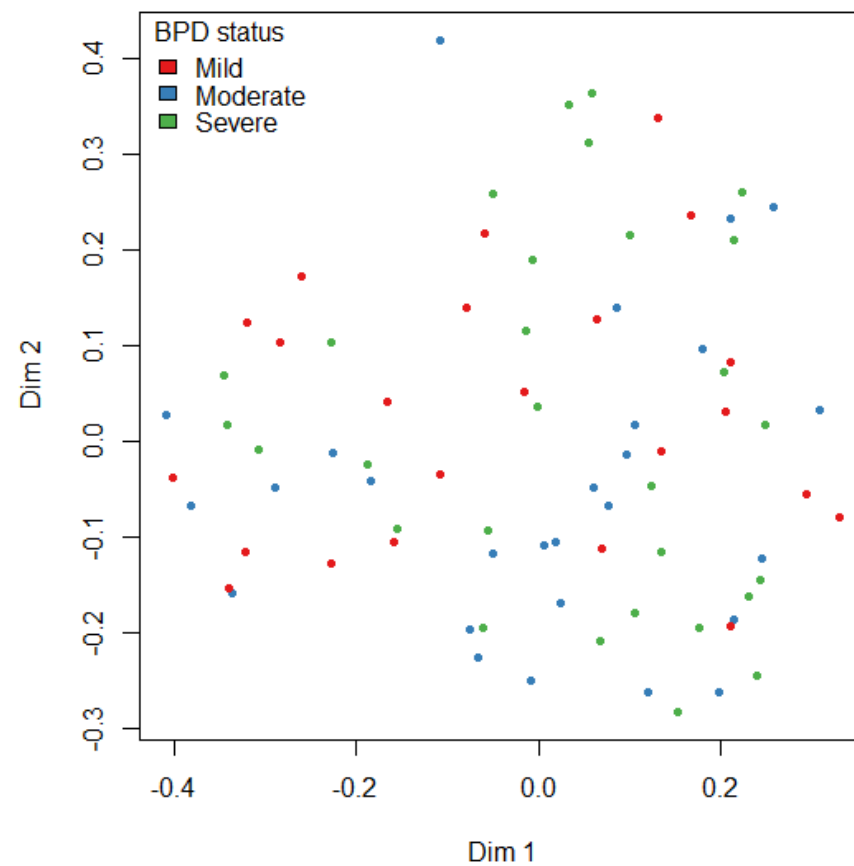

Supplement: S1 Fig — Multidimensional Scaling plot using the proximity matrix from the RF shows very little separation between the BPD groups which corresponds with the high error rate (right). (PDF) [file pone.0170120.s005.pdf]

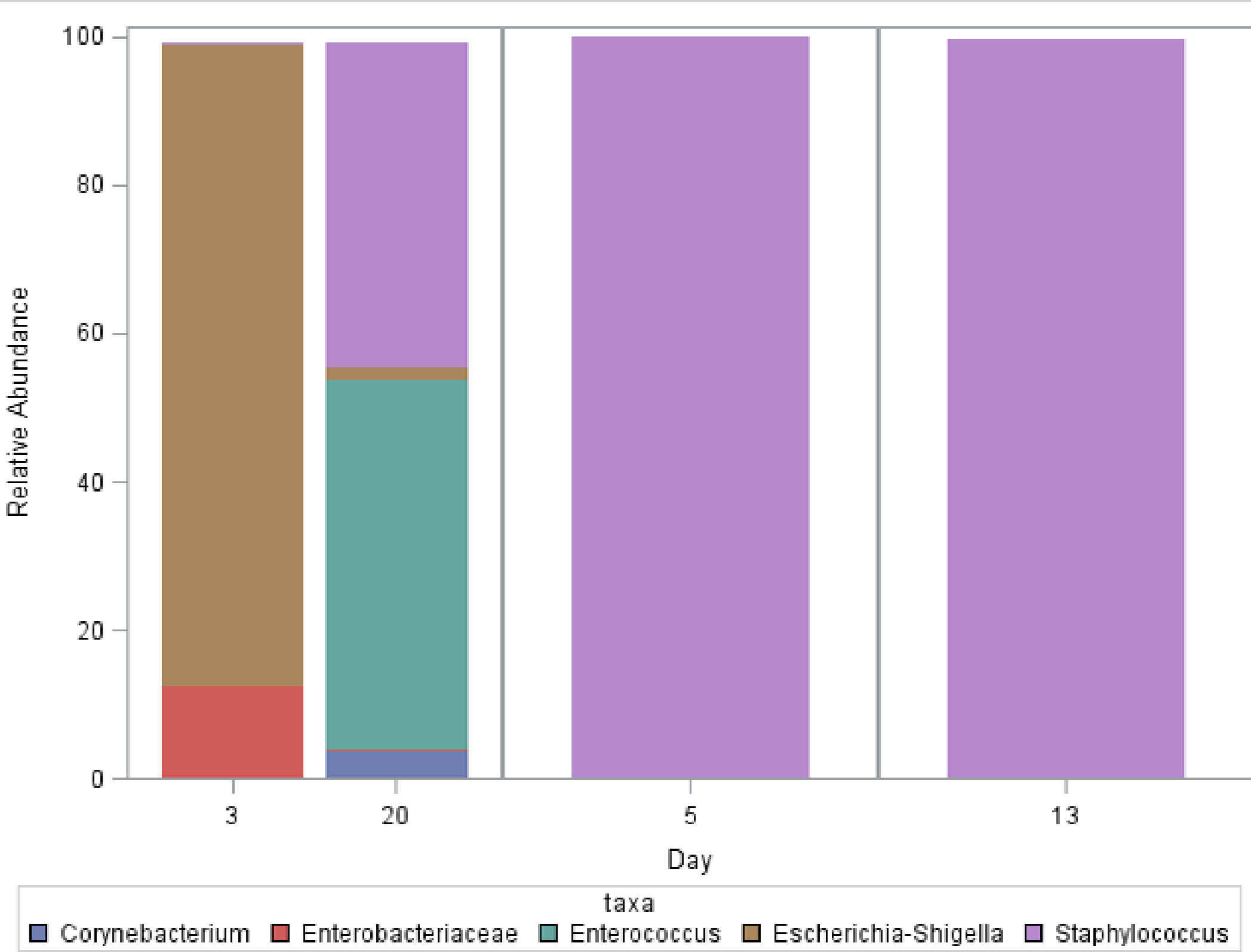

Supplement: S2 Fig — (PDF) [file pone.0170120.s006.pdf]

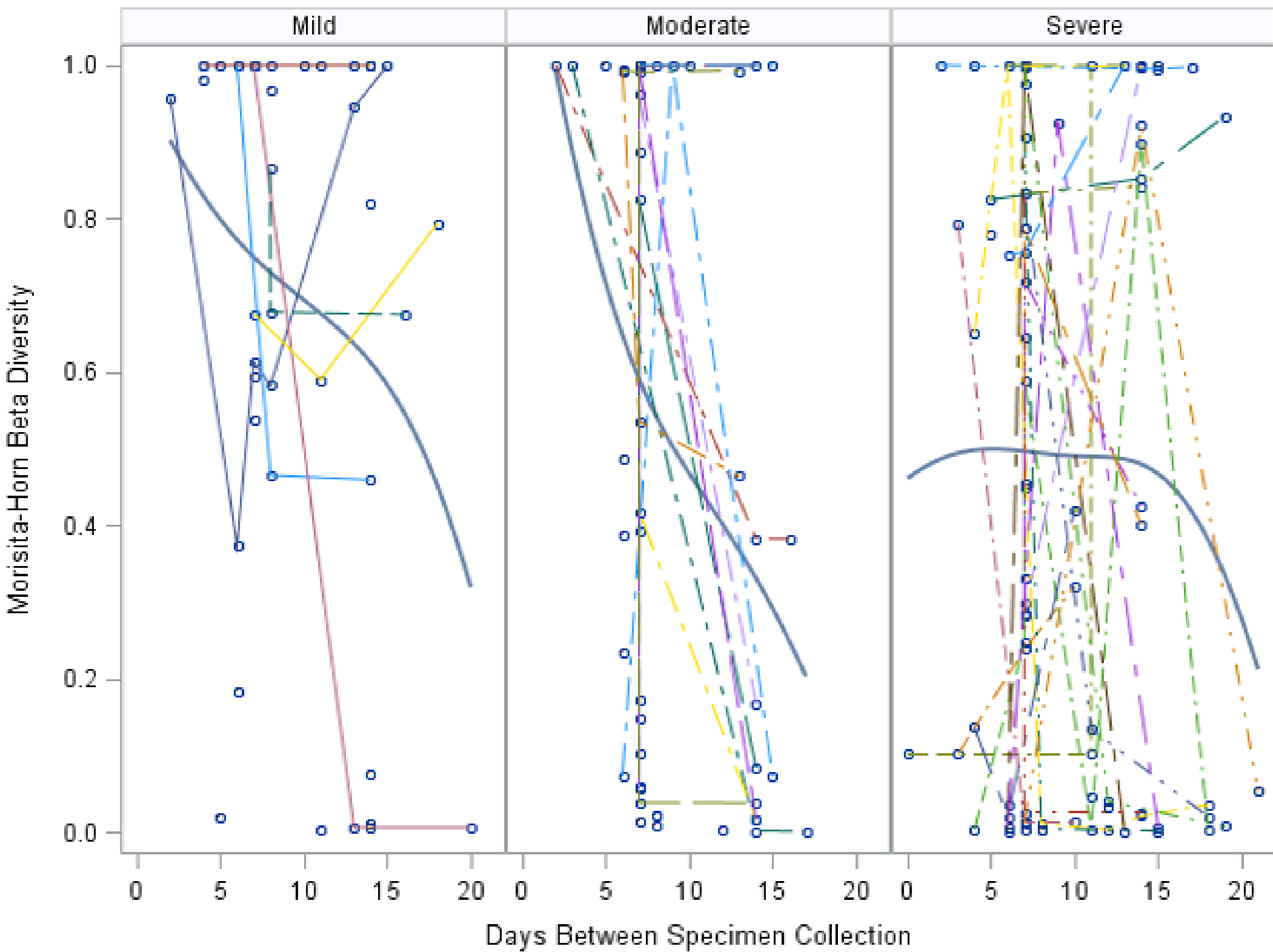

Supplement: S3 Fig — There is a trend towards greater community turnover in the moderate and severe BPD groups (Mild n = 25, Moderate N = 39 and Severe N = 30). The bold blue line indicates the average trend. (PDF) [file pone.0170120.s007.pdf]
